# A novel inflammatory biomarker, GlycA, associates with disease activity in rheumatoid arthritis and cardio-metabolic risk in BMI-matched controls

**DOI:** 10.1186/s13075-016-0982-5

**Published:** 2016-04-12

**Authors:** David B. Bartlett, Margery A. Connelly, Hiba AbouAssi, Lori A. Bateman, K. Noelle Tune, Janet L. Huebner, Virginia B. Kraus, Deborah A. Winegar, James D. Otvos, William E. Kraus, Kim M. Huffman

**Affiliations:** Department of Medicine and Duke Molecular Physiology Institute, Duke School of Medicine, Durham, NC USA; LipoScience, Laboratory Corporation of America® Holdings, Raleigh, NC USA; Center for Health Promotion and Disease Prevention, University of North Carolina at Chapel Hill, Chapel Hill, NC USA

**Keywords:** Rheumatoid arthritis, Inflammation, Biomarker, Metabolic syndrome, Glycosylation

## Abstract

**Background:**

RA and CVD both have inflammation as part of the underlying biology. Our objective was to explore the relationships of GlycA, a measure of glycosylated acute phase proteins, with inflammation and cardiometabolic risk in RA, and explore whether these relationships were similar to those for persons without RA.

**Methods:**

Plasma GlycA was determined for 50 individuals with mild-moderate RA disease activity and 39 controls matched for age, gender, and body mass index (BMI). Regression analyses were performed to assess relationships between GlycA and important markers of traditional inflammation and cardio-metabolic health: inflammatory cytokines, disease activity, measures of adiposity and insulin resistance.

**Results:**

On average, RA activity was low (DAS-28 = 3.0 ± 1.4). Traditional inflammatory markers, ESR, hsCRP, IL-1β, IL-6, IL-18 and TNF-α were greater in RA versus controls (P < 0.05 for all). GlycA concentrations were significantly elevated in RA versus controls (P = 0.036). In RA, greater GlycA associated with disease activity (DAS-28; R_DAS-28_ = 0.5) and inflammation (R_ESR_ = 0.7, R_hsCRP_ = 0.7, R_IL-6_ = 0.3: P < 0.05 for all); in BMI-matched controls, these inflammatory associations were absent or weaker (hsCRP), but GlycA was related to IL-18 (R_hsCRP_ = 0.3, R_IL-18_ = 0.4: P < 0.05). In RA, greater GlycA associated with more total abdominal adiposity and less muscle density (R_abdominal-adiposity_ = 0.3, R_muscle-density_ = −0.3, P < 0.05 for both). In BMI-matched controls, GlycA associated with more cardio-metabolic markers: BMI, waist circumference, adiposity measures and insulin resistance (R = 0.3-0.6, P < 0.05 for all).

**Conclusions:**

GlycA provides an integrated measure of inflammation with contributions from traditional inflammatory markers and cardio-metabolic sources, dominated by inflammatory markers in persons with RA and cardio-metabolic factors in those without.

## Background

Rheumatoid arthritis (RA) is a chronic inflammatory autoimmune disease that, when left uncontrolled, leads to debilitating alterations in joint function. Therefore, it is not uncommon for patients with RA to be physically inactive, leading to increased adiposity, body mass index (BMI), and insulin resistance [1]. Recently, we showed that reduced skeletal muscle insulin sensitivity in RA patients is more likely due to traditional metabolic risk factors such as adiposity than to systemic inflammation or disease-related factors [2]. Given the multiple potential contributors to progression to type 2 diabetes mellitus (T2DM) and known increased prevalence (2–3-fold) of cardiovascular disease (CVD) in RA, a holistic biomarker of risk of these conditions would be extremely useful for targeting appropriate early preventive and treatment strategies [3–6].

GlycA is a marker of inflammation measured by nuclear magnetic resonance (NMR) spectroscopy that has been shown to be associated with cardiometabolic disease and mortality [7–12]. The GlycA NMR signal arises largely from the *N*-acetyl glucosamine residues on the carbohydrate side-chains of acute phase proteins such as α_1_-acid glycoprotein, α_1_-antitrypsin, α_1_-antichymotrypsin, haptoglobin, and transferrin [7]. This composite NMR signal, termed “GlycA,” has been shown to be strongly associated with both incident CVD and incident T2DM in the Women’s Health Study (WHS) and the Prevention of Renal and Vascular End-stage Disease study (PREVEND) as well as with all-cause mortality in the WHS and Justification for the Use of Statin in Prevention: An Intervention Trial Evaluating Rosuvastatin (JUPITER), even after adjusting for traditional risk factors [8–10]. Recently, GlycA was elevated compared with controls and related to RA disease activity and coronary calcium scores in persons with RA [13] as well as in patients with systemic lupus erythematosus [14]. With this in mind, we sought to better understand whether GlycA was associated with markers of inflammation and cardiometabolic risk in a cohort of RA patients who were extensively characterized for disease activity, adiposity, and insulin sensitivity.

## Methods

### Participants and design

The study design and procedures have been reported previously [2]. Briefly, this study was designed as a cross-sectional comparison of insulin sensitivity between persons with RA and controls matched for age (±3 years), sex, race, and BMI (±3 kg/m^2^). Persons with RA were either seropositive or had erosions on radiographs, met 1987 American College of Rheumatology criteria for RA [15], had no medication changes in the last 3 months, and were using stable doses of prednisone of 5 mg per day or less. Exclusions were known diabetes mellitus or CVD. A total of 50 subjects with RA and 39 matched controls were recruited consecutively and included in this study. All participants signed an informed consent. The study was approved by the Duke University Medical Center Institutional Review Board.

### Assessments

We previously described methods for determining disease activity (Disease Activity Score with 28-joint count using the erythrocyte sedimentation rate (DAS_ESR_-28)), pain (visual analog scale), disability (Health Assessment Questionnaire—Disability Index (HAQ-DI)), insulin sensitivity indices from frequently sampled intravenous glucose tolerance tests (IVGTTs), and fasting glucose, insulin, and inflammatory marker concentrations [2]. Abdominal and thigh adipose depots were determined as described previously [2] using single 10-mm-thick axial computed tomography (CT) scan sections in the liver, mid-abdomen at L4, and mid-thigh (General Electric CT/I scanner; GE Medical Systems, Milwaukee, WI, USA).

### GlycA measurements

NMR spectra were acquired from ethylenediaminetetraacetic acid plasma samples as described previously for the NMR LipoProfile® (lipoprotein particle) test at LipoScience (now LabCorp, Raleigh, NC, USA) [16]. The GlycA NMR signal (2.00 ± 0.01 ppm) was quantified as described previously, using a proprietary software algorithm [17]. Briefly, the NMR signal amplitudes originate from highly mobile *N*-acetyl methyl group protons of the *N*-acetylglucosamine moieties located on the carbohydrate side-chains of circulating plasma proteins (predominantly α_1_-acid glycoprotein, haptoglobin, α_1_-antitrypsin, α_1_-antichymotrypsin, and transferrin) were used to calculate the concentrations of GlycA (in μmol/l of *N*-acetyl methyl groups). The intra-assay and inter-assay variability for GlycA measurement is 1.9 % and 2.6 %, respectively [7].

### Statistical analyses

All analyses were conducted using SAS 9.4 (SAS Institute Inc., Cary, NC, USA) except for Fisher transformations. Strengths of GlycA associations for the two groups (RA and controls) were compared with Fisher *r* to *z* transformations, computed using an online calculator [17]. Normality was assessed with Kolmogorov–Smirnov goodness of fit testing. Differences between groups were assessed by either independent *t* tests or Mann–Whitney nonparametric tests depending on normality. Bivariate associations were assessed with Spearman correlations. Non-normally distributed variables with significant correlations were logarithmically transformed and multivariable modeling was performed using linear models with forward stepwise selection. Significance was accepted at *P* <0.05.

## Results

**Fig. 1:**
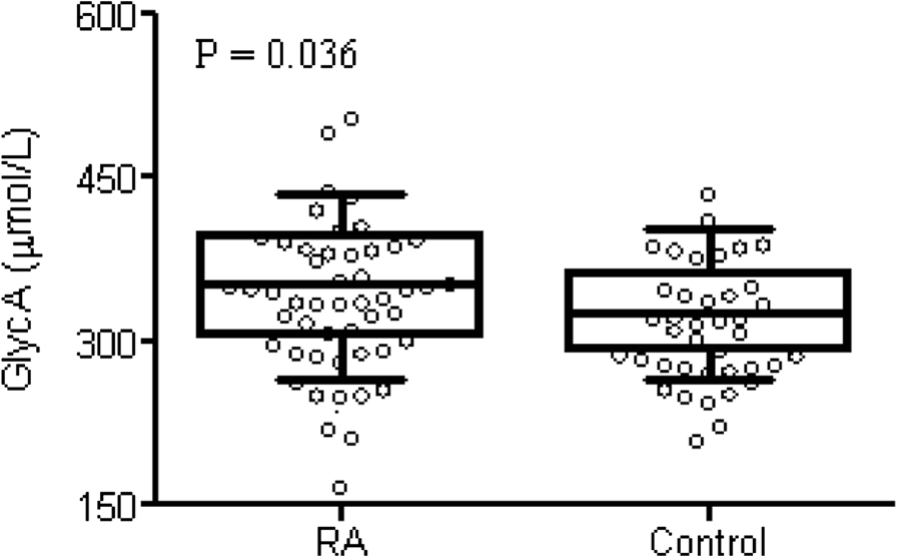
GlycA is greater in patients with mild–moderate RA compared with BMI-matched control subjects. *Boxes* represent the mean (*middle horizontal line*) and the 25th and 75th percentiles. *Whiskers* represent the 10th and 90th percentiles. Each data point is presented as an *open circle*. Mean ± standard deviation concentration of GlycA was greater in persons with RA (352.8 ± 67.2) than in controls (328.9 ± 53.5); *P* = 0.036 using an independent *t* test. *RA* rheumatoid arthritis

Participants were matched for age, gender, and BMI, and thus no differences were observed for measures of cardiometabolic risk including adiposity (*P* >0.05 for all), except for fasting glucose which was slightly lower in RA patients (*P* = 0.018). As reported previously, persons with RA had a range of disease activity (DAS_ESR_-28 range = 0.6–6.4), but on average disease activity was mild to moderate (mean ± standard deviation DAS_ESR_-28 = 3.0 ± 1.4) [2]. As expected, measures of inflammation, erythrocyte sedimentation rate (ESR), high-sensitivity C-reactive protein (hsCRP), interleukin (IL)-1β, IL-6, IL-18, and tumor necrosis factor alpha (TNFα) concentrations were greater in persons with RA as compared with matched controls (Table 1; *P* <0.05 for all) while IL-8 was lower (Table 1; *P* = 0.027). GlycA concentrations were greater in persons with RA than matched controls (Fig. 1; GlycA 352.8 ± 67.2 vs. 328.9 ± 53.5 μmol/l, *P* = 0.036).

**Table 1:** Participant demographics, clinical characteristics, and inflammation

	Rheumatoid arthritis (*n* = 50)	Controls (*n* = 39)
Age (years)	55.4 ± 12.8	52.1 ± 11.4
Gender		
Female	35 (70 %)	27 (69 %)
Male	15 (30 %)	12 (31 %)
Race		
Pacific Islander	1 (2 %)	0 (0 %)
African American	14 (28 %)	12 (31 %)
Caucasian	35 (70 %)	27 (69 %)
Clinical characteristics		
BMI (kg/m^2^)	30.5 ± 7.5	29.0 ± 5.3
Waist circumference (cm)	95.3 ± 16.7	85.0 ± 27.9
HAQ-DI	0.7 ± 0.7***	0 ± 0
Pain (VAS) (mm)	40.1 ± 28.9***	9.8 ± 2.4
Comorbidity index	1.6 ± 1.2**	0.6 ± 0.9
DAS_ESR_-28	3.0 ± 1.4	NA
Remission (DAS <2.6)	19 (40 %)	
Low activity (DAS 2.6–3.2)	8 (17 %)	
Moderate activity (DAS 3.2–5.1)	16 (33 %)	
High activity (DAS >5.1)	5 (10 %)	
RF positive	41/46	NA
Anti-CCP positive	20/21	NA
Radiograph erosions present	21/38	NA
Medication use		
Etanercept	10 (20 %)	NA
Infliximab	2 (4 %)	NA
Adalimumab	5 (10 %)	NA
Abatacept	5 (10 %)	NA
Methotrexate	38 (76 %)	NA
Leflunomide	1 (2 %)	NA
Sulfasalazine	0	NA
Hydroxychloroquine	10 (20 %)	NA
NSAID	18 (36 %)	NA
Prednisone	12 (24 %)	NA
Systemic inflammation (mean ± SEM)		
ESR (mm/hour)	11.9 ± 1.7*	7.6 ± 2.6
hsCRP (mg/l)	7.9 ± 1.2**	3.2 ± 0.7
IL-1β (pg/ml)	0.8 ± 0.2*	0.7 ± 0.2
IL-6 (pg/ml)	19.8 ± 7.3***	3.1 ± 0.3
IL-8 (pg/ml)	10.9 ± 1.1*	17.8 ± 8.0
IL-18 (pg/ml)	464.2 ± 21.1**	390.8 ± 21.8
TNFα (pg/ml)	31.5 ± 5.0***	11.4 ± 8.6
Metabolic		
Fasting insulin (mU/l)	7.5 ± 7.5	7.5 ± 5.6
Fasting glucose (mg/dl)	89.9 ± 13.6*	97.2 ± 11.4
HOMA	1.7 ± 1.8	1.8 ± 1.5
IS index (×10^–5.^min^−1^/(pmol/l))		
Women	6.8 ± 6.5	8.4 ± 10.4
Men	4.2 ± 3.3	5.3 ± 3.3
Acute insulin response (pmol/l)	481 ± 523	324 ± 230
Adiposity and muscle		
Abdominal		
Total adipose area (cm^2^)	411 ± 201	400 ± 157
Subcutaneous adiposity (cm^2^)	306 ± 155	275 ± 132
Visceral adiposity (cm^2^)	105 ± 86	125 ± 96
Liver density (Hu)	60 ± 11	59 ± 12
Thigh		
Total thigh area (cm^2^)	250 ± 74	244 ± 59
Subcutaneous adiposity (cm^2^)	124 ± 63	108 ± 51
Intermuscular adiposity (cm^2^)	12 ± 7	12 ± 8
Muscle area (cm^2^)	115 ± 37	124 ± 32
Muscle density (Hu)	51 ± 6	52 ± 4

Data presented as mean ± standard deviation or frequency (percentage) unless otherwise stated

*P <0.05, **P <0.01, ***P <0.001 as compared with controls

*BMI* body mass index, *CCP* cyclic citrullinated peptide, *DAS* Disease Activity Score, *DAS*_*ESR*_ *-28* Disease Activity Score with 28-joint count using the erythrocyte sedimentation rate, *ESR* erythrocyte sedimentation rate, *HAQ-DI* Health Assessment Questionnaire—Disability Index, *HOMA* homeostasis model assessment, *hsCRP* high-sensitivity C-reactive protein, *IL* interleukin, *IS* insulin sensitivity, *NSAID* nonsteroidal anti-inflammatory drug, *RF* rheumatoid factor, *SEM* standard error of the mean, *TNFα* tumor necrosis factor alpha, *VAS* visual analog scale, *NA* not applicable

Among persons with RA (*n* = 50), GlycA concentrations were related positively to ESR (*r* = 0.71, *P* <0.001), hsCRP (*r* = 0.73, *P* <0.001), and disease activity (DAS_ESR_-28; *r* = 0.54, *P* <0.001), but not to pain or disability (Table 2). Of other circulating markers of inflammation, GlycA was related to IL-6 (*r* = 0.28, *P* <0.05) but not to IL-1β, IL-8, IL-18, or TNFα. Positive associations were observed between GlycA and total abdominal adiposity (*r* = 0.31, *P* <0.04) and fasting glucose (*r* = 0.35, *P* <0.01) while less thigh muscle density was associated with more GlycA (*r* = −0.33, *P* <0.02). In a multivariable model for persons with RA, hsCRP, ESR, and thigh muscle density were each related independently to GlycA, and together explained 74 % of the variance in GlycA (*P* <0.001, *R*_hsCRP_ = 0.59, *R*_ESR_ = 0.11, *R*_thigh muscle density_ = 0.05; Table 3).

**Table 2:** GlycA relationships with disease activity, inflammatory, and adiposity measures

	GlycA		Fisher transformation
	RA	Controls	*P* value
Markers of disease activity and inflammation			
Disability (HAQ-DI)	0.28	NA	NA
Pain (VAS) (mm)	0.08	0.11	0.91
Disease activity (DAS_ESR_-28)	**0.54** ^**‡**^	NA	NA
ESR (mm/hour)	**0.71** ^**‡**^	0.24	**0.02**
hsCRP (mg/l)	**0.73** ^**‡**^	**0.32** ^**†**^	**0.01**
IL-1β (pg/ml)	0.19	0.08	0.62
IL-6 (pg/ml)	**0.28** ^**†**^	−0.14	0.05
IL-8 (pg/ml)	−0.20	−0.07	0.56
IL-18 (pg/ml)	−0.14	**0.41** ^**†**^	**0.01**
TNFα (pg/ml)	−0.09	−0.14	0.82
Measures of adiposity and insulin resistance			
BMI (kg/m^2^)	0.16	**0.38** ^**†**^	0.28
Waist circumference (cm)	0.12	**0.38** ^**†**^	0.22
Total abdominal adiposity (cm^2^)	**0.31** ^**†**^	**0.36** ^**†**^	0.80
Abdominal subcutaneous adiposity (cm^2^)	0.26	**0.34** ^**†**^	0.70
Abdominal visceral adiposity (cm^2^)	0.25	0.21	0.86
Thigh subcutaneous adiposity (cm^2^)	0.21	**0.55** ^**‡**^	0.08
Thigh intermuscular adiposity (cm^2^)	0.06	0.18	0.60
Thigh muscle density (Hu)	**−0.33** ^**†**^	−0.14	0.40
Fasting insulin (mU/l)	0.18	**0.37** ^**†**^	0.38
Fasting glucose (mg/dl)	**0.35** ^**†**^	0.16	0.36
HOMA	0.17	**0.39** ^**†**^	0.30
IS index (×10^–5.^min^−1^/(pmol/l))	−0.26	**−0.34** ^**†**^	0.70
Acute insulin response (pmol/l)	0.05	**0.48** ^**†**^	**0.04**

Data presented as Spearman correlation coefficients (*r*)

^†^0.005 < *P* <0.05, ^‡^ *P* ≤0.005

*BMI* body mass index, *DAS*_*ESR*_ *-28* Disease Activity Score with 28-joint count using the erythrocyte sedimentation rate, *ESR* erythrocyte sedimentation rate, *HAQ-DI* Health Assessment Questionnaire–Disability Index, *HOMA* homeostasis model assessment, *hsCRP* high-sensitivity C-reactive protein, *IL* interleukin, *IS* insulin sensitivity, *RA* rheumatoid arthritis, *TNFα* tumor necrosis factor alpha, *VAS* visual analog scale, *NA​* not applicable

**bold** text represents significant associations

**Table 3:** Multivariable models for GlycA (log) in persons with rheumatoid arthritis and controls

	Parameter estimate	Partial *R* ^2^	*P* value
Rheumatoid arthritis: model *R* ^2^ = 0.74, *n* = 48			
hsCRP (log mg/l)	58.4	0.59	<0.0001
ESR (mm/hour)	1.8	0.11	0.002
Thigh muscle density (Hu)	−2.7	0.05	0.01
Controls without rheumatoid arthritis: model *R* ^2^ = 0.47, *n* = 33			
Thigh subcutaneous adiposity (cm^2^)	0.54	0.35	0.0003
IL-18 (log pg/ml)	132.6	0.12	0.01

Multivariable modeling was performed using linear models with forward stepwise variable selection. Variables for forward selection were based on significant results from bivariate analyses shown in Table 2. For RA patients, variables were selected from disease activity (DAS-28), ESR, hsCRP (log), IL-6 (log), total abdominal adiposity, thigh muscle density, fasting glucose, age, and gender. For controls, variables were selected from hsCRP (log), IL-18 (log), total abdominal adiposity, thigh subcutaneous adiposity, acute insulin response, insulin sensitivity, age, and gender. Final models are shown

*DAS-28* Disease Activity Score, *ESR* erythrocyte sedimentation rate, *hsCRP* high-sensitivity C-reactive protein, *IL* interleukin

Among controls (*n* = 39), GlycA concentrations were positively related to hsCRP (*r* = 0.32, *P* <0.05) and IL-18 (*r* = 0.41, *P* <0.01). GlycA was also related to multiple measures of adiposity, including BMI (*r* = 0.38, *P* <0.02), waist circumference (*r* = 0.38, *P* <0.02), total abdominal adiposity (*r* = 0.36, *P* <0.03), and abdominal (*r* = 0.34, *P* <0.04) and thigh (*r* = 0.55, *P* <0.001) subcutaneous adiposity. GlycA was associated with measures of insulin resistance including fasting insulin (*r* = 0.37, *P* <0.02), homeostasis model assessment (HOMA; *r* = 0.39, *P* <0.02), and insulin sensitivity (*r* = −0.34, *P* <0.04). In a multivariable model for controls without RA, both thigh subcutaneous adiposity and IL-18 were related independently to GlycA, and together explained 47 % of the variance in GlycA (*P* <0.001, *R*_thigh subcutaneous adiposity_ = 0.35, *R*_IL-18_ = 0.12; Table 3). GlycA was not related to age or sex in either RA or non-RA controls (*r* <0.13 for all).

The GlycA associations strengths were different between persons with RA and controls for ESR, hsCRP, IL-18, and acute insulin response to glucose (Table 2; *P* <0.05 for all). In persons with RA, GlycA was more strongly related to the inflammatory markers ESR and hsCRP, while in controls GlycA was more strongly related to IL-18 and acute insulin response to glucose.

## Discussion

In this study, GlycA concentrations and associations were compared between mild to moderately active persons with RA and controls matched for age, sex, and BMI. GlycA concentrations were greater for those with RA. Further, GlycA associations differed between the groups for measures of inflammation (ESR, hsCRP, IL-18) and insulin sensitivity (acute insulin response to glucose). In the absence of RA, GlycA concentrations reflected cardiometabolic risks of adiposity and reduced insulin sensitivity. In persons with RA, GlycA reflected primarily disease activity-related inflammation.

Although IL-6 and TNF are associated with RA pathology, the molecular mechanism of the disease pathology remains unknown. Furthermore, commonly used measures of RA disease severity, CRP, and ESR are nonspecific, with increased concentrations observed in other chronic conditions and obesity [18–20]. Identification of a novel inflammatory biomarker representative of disease-specific activity is therefore critical to identifying new treatments and targets of RA. We show here for the first time that GlycA is greater in RA and is predominantly associated with typical systemic inflammation and less so with adiposity.

The GlycA signal arises largely from the carbohydrate side-chains on acute phase proteins. Most circulating acute phase proteins are N-linked glycoproteins. Both acute inflammation and chronic inflammation induce synthesis and secretion of increased amounts of these glycoproteins. Further, inflammation produces increased protein glycosylation and glycan structure branching [21–23]. All of these glycan modifications lead to increases in GlycA signals. While RA pathogenesis involves IL-6-driven upregulation of the acute phase response [24], IL-6, CRP, and fibrinogen contribute negligibly, if at all, to the GlycA signal [7]. Instead, for the GlycA signal the main contributors are the acute phase proteins α_1_-acid glycoprotein, α_1_-antitrypsin, α_1_-antichymotrypsin, transferrin, and haptoglobin [7]. These acute phase proteins serve as regulators of inflammation, are expressed more in RA, and contribute to RA pathogenesis [25]. Thus, in RA, increased inflammation drives increases in concentrations and glycosylation of acute phase proteins leading to increased GlycA.

In addition to amounts of GlycA, RA-specific associations for GlycA suggest differences in GlycA composition. In RA, GlycA may contain different acute phase protein glycosylations, isoforms, and/or proportions. For example, haptoglobin is a hemoglobin binding protein responsible for limiting tissue damage caused by hemoglobin-induced oxidative stress [26, 27]. While haptoglobin is typically anti-inflammatory, glycosylation site alterations have been identified in RA and other diseases such as cancer; the ability of glycosylation to alter protein function and immunogenicity suggests that glycosylation alterations may serve pathogenic roles [28–30]. In RA synovial fluid, a specific haptoglobin isoform upregulates monocyte IL-6 production [31]. Also, synthesis of haptoglobin is primarily hepatic; however, it is also produced by activated neutrophils and taken up peripherally by monocytes [32, 33]. Thus, the source, balance, and functions of haptoglobin and other acute phase proteins in RA are different from those in healthy controls and likely contribute to different GlycA associations [31]. Although we did not assess the individual acute phase protein contributions to GlycA in this sample, we suggest that GlycA is a comprehensive measure of pathogenic inflammation in RA.

The full clinical implications of GlycA in RA are thus unclear. Given that it reflects multiple types of inflammation, GlycA may be able to serve as a composite marker of overall inflammatory risk in RA. An example is the work showing that GlycA was associated with coronary artery calcium in RA [13]. It is likely that both disease-related and adiposity-related inflammation contribute to RA cardiovascular risk as well as other negative outcomes. Future work is necessary to define the role of GlycA in RA early preventive and treatment strategies.

In those without RA, the GlycA signal appears to be driven by glycosylation of a different set of acute phase proteins, those associated with cardiometabolic risk [13, 34]. Recently, GlycA was associated with greater leptin to adiponectin ratios [34], an indicator of dysfunctional adipose tissue, leptin resistance, and insulin resistance, in subjects with metabolic syndrome or type 2 diabetes [35, 36]. Here, greater GlycA concentrations were associated with more adiposity as reflected by larger BMIs, larger waist circumferences, and greater amounts of thigh and abdominal subcutaneous adiposity. Also, greater GlycA levels, but not hsCRP (data not shown), were associated with more fasting insulin, more pancreatic beta-cell insulin secretion, and less skeletal muscle insulin sensitivity; all indicators of greater diabetes risk.

GlycA was associated with increased IL-18 in those without RA but not in those with RA, again highlighting differences in inflammation associated with chronic inflammatory diseases and obesity. In RA, IL-18 concentrations are greater than those without RA and are related to disease activity [37, 38]. IL-18 acts locally within the synovium to stimulate macrophage production of TNFα; subsequently, TNFα stimulates synovial fibroblast production of IL-18, generating a positive, inflammatory feedback loop [37]. IL-18 stimulates fibroblasts to secrete mediators of leukocyte recruitment and activation, angiogenesis, and cartilage destruction [37].

While IL-18 is secreted primarily by macrophages and other immune cells, adipocytes are capable of constitutively producing IL-18 and increase IL-18 synthesis in obesity [39, 40]. IL-18 has been shown to be a marker of metabolic disease, insulin resistance, and CVD risk, and is reduced following exercise and diet [40, 41]. Perhaps adipose tissue-derived, but not synovial-derived or immune cell-derived, IL-18 leads to altered acute phase protein glycosylation, but additional work is necessary to confirm this assertion.

We recognize that this study has several limitations. While performing multiple correlations increased the possibility for type I statistical errors, we attempted to minimize the likelihood by integrating the findings into themes (i.e. traditional inflammation and cardiometabolic risk) of associations for GlycA. Also, while the sample size is limited, we believe this is outweighed by the strength of detailed phenotyping with CT scans for adiposity measures and IVGTTs for insulin action. Additionally, as this investigation is cross-sectional, causal relationships cannot be proven. Most importantly, this study is unable to comment on how GlycA levels might change over time with changes in disease activity or cardiometabolic risks.

## Conclusions

In summary, GlycA provides an integrated measure of inflammation with contributions from traditional inflammatory and cardiometabolic sources, dominated by the former in persons with RA and by the latter in those without. Taken together, these findings suggest that the glycosylation mechanism of acute phase proteins is different in inflammatory disease compared with increased adiposity. Additional investigations, especially longitudinal studies, will illuminate roles for GlycA to serve as a biomarker for inflammatory and cardiometabolic disease.

## Abbreviations

BMI: Body mass index
CT: Computed tomography
CVD: Cardiovascular disease
DAS_ESR_-28: Disease Activity Score with 28-joint count using the erythrocyte sedimentation rate
ESR: Erythroctye sedimentation rate
HAQ-DI: Health Assessment Questionnaire—Disability Index
HOMA: Homeostasis model assessment
hsCRP: High-sensitivity C-reactive protein
IL: Interleukin
IS: insulin sensitivity
IVGTT: Intravenous glucose tolerance test
NMR: Nuclear magnetic resonance
PREVEND: Prevention of Renal and Vascular End-stage Disease study
RA: Rheumatoid arthritis
T2DM: Type 2 diabetes mellitus
TNFα: Tumor necrosis factor alpha
WHS: Women’s Health Study
